# Functionalization of a Volume-Stable Collagen Matrix Using Liquid Platelet-Rich Fibrin: A Case Report Presenting a New Approach for Root Coverage

**DOI:** 10.1155/2023/3929269

**Published:** 2023-03-22

**Authors:** Roberta Michels, Gabriel Leonardo Magrin, ACC Cruz, RS Magini, Cesar Augusto Magalhães Benfatti

**Affiliations:** Center for Education and Research on Dental Implants (CEPID), Post-Graduate Program in Dentistry (PPGO), Federal University of Santa Catarina (UFSC), Florianópolis, Brazil

## Abstract

This case report presents a novel approach for root coverage of multiple gingival recessions with a volume-stable collagen matrix functionalized with injectable platelet-rich fibrin (i-PRF). A patient with multiple gingival recessions in the anterior maxilla was submitted to root coverage by coronally advanced flap with split–full–split incisions. Blood collection was performed before surgery and i-PRF was obtained after centrifugation (relative centrifugal force (RCF) 400 g, 2700 rpm, 3 minutes). A volume-stable collagen matrix was soaked with i-PRF and applied as a substitute for autogenous connective tissue graft. A mean root coverage of 83% was observed after a 12-month follow-up period, and only slight modifications were detected in a 30-month follow-up consultation. The association of a volume-stable collagen matrix with i-PRF successfully treated multiple gingival recessions with reduced morbidity since a connective tissue collection was avoided.

## 1. Introduction

Gingival recession is a condition that can be defined as the apical migration of gingival tissue from the cementoenamel junction and is associated with the loss of supportive periodontium [1]. Consequently, root exposure to the oral environment is observed, and patients can present dentin hypersensitivity, root caries, and aesthetics complains [2]. Gingival recession can be caused by several factors like traumatic toothbrushing, inadequate tooth position (i.e., crowded teeth), orthodontic movement, deep cervical restorative margins, and persistent gingival inflammation [3]. Root coverage techniques have been employed to treat gingival recessions. According to the interproximal involvement, this condition can be classified as: RT1—no interproximal insertion loss; RT2—interproximal insertion loss not higher than the buccal level; and RT3—interproximal insertion loss higher than the buccal level [4]. The best prognosis has been observed in RT1 lesions [4, 5].

Coronally advanced flap (CAF) associated with autogenous connective tissue graft has demonstrated long-term effectiveness in multiple gingival recession treatment [6]. However, autogenous graft collection has limitations such as the risk of hemorrhage, limited availability of donor tissue, anatomical variations between patients, and postoperative morbidity [7]. Taking those points into consideration, alternative biomaterials have been developed for root coverage with stable outcomes and less invasive techniques, such as acellular dermal matrix, xenogeneic collagen matrix, enamel matrix derivative, and platelet aggregates [8, 9]. Xenogeneic collagen matrix has been used as a substitute for autogenous connective tissue graft; however, while autogenous tissue has viable fibroblasts and signaling molecules for stable long-term outcomes when associated with root coverage procedures [10, 11], xenogeneic matrices only mimic the extracellular matrix and do not have a robust evidence in terms of root coverage results [12]. In this context, adding signaling molecules into collagen matrices may increase cell migration, adhesion, and differentiation, improving patient healing and the overall root coverage result, as suggested in observations of a proof-of-concept study with acellular dermal matrix allografts [13].

Platelet-rich fibrin (PRF) is a blood-derived reservoir of bioactive molecules that are known to promote wound healing and tissue regeneration [14]. This platelet concentrate can act as a delivery system of molecules such as transforming growth factor (TGF)-*β* and collagen type 1, which increases the release of other growth factors and signaling proteins [15]. Moreover, the PRF has been extensively studied in periodontology due to the potential for keratinization in surgical sites [16, 17]. Studies reported that injectable PRF (i-PRF) stimulates more vascularization and cell chemotaxis than other PRF preparations [15, 18]. Therefore, the aim of this case report was to describe a multiple gingival recession case treated by CAF associated with a volume-stable collagen matrix functionalized with i-PRF.

## 2. Case Presentation

### 2.1. Baseline Examination

**Figure 1:**
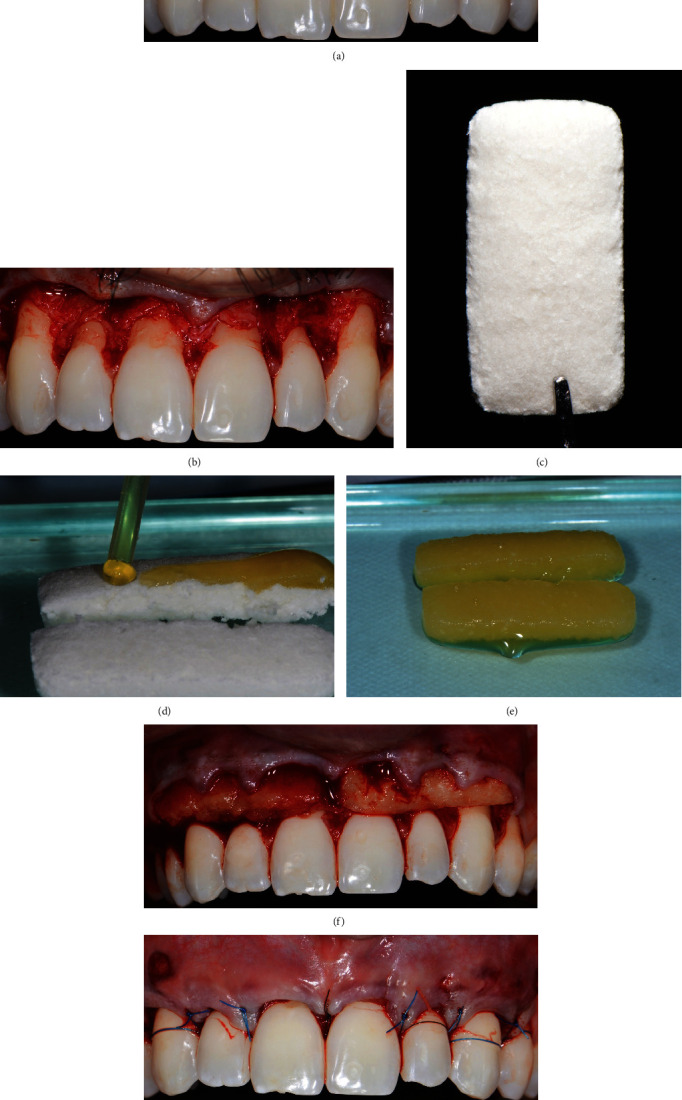
Preoperative evaluation and surgical procedure. (a) Clinical view before surgery, (b) a surgical flap with a split–full–split thickness configuration was raised, (c) volume-stable collagen matrix (Fibro-Gide, Geistlich Pharma AG, Wolhusen, Switzerland) before platelet-rich fibrin functionalization, (d) functionalization of the collagen matrix with injectable platelet-rich fibrin (i-PRF), (e) collagen matrix after functionalization, (f) positioning of the matrix impregnated with injectable platelet-rich fibrin (i-PRF) in the surgical site, (g) tension-free flap repositioning and sutures.

A 26-year-old male patient sought the Center for Education and Research on Dental Implants at the Federal University of Santa Catarina (CEPID/UFSC) in February 2020, due to multiple gingival recessions in the aesthetic area of the maxilla caused by orthodontic treatment and brushing trauma. During clinical examination, a thin gingival phenotype was detected (Figure 1(a)). Since no interproximal involvement was present, the gingival recessions were classified as RT1 [4]. Probing depths were ≤3 mm for all periodontal sites, bleeding on probing was present in 27.78% of the sites, and plaque index was 15%. The patient was diagnosed with biofilm-induced gingivitis [19].

### 2.2. Ethical Aspects

The patient was informed about the characteristics of the intervention and the data collection both orally and through a written information sheet. An informed consent was signed by the patient. No institutional approval of the ethics committee was necessary for this case report.

### 2.3. Preoperative Clinical Sessions

Before the surgical procedure, gingival health was obtained. The patient received brushing technique instructions, and gingivitis was treated with non-surgical periodontal therapy involving ultrasonic debridement, root planning, and professional prophylaxis. The patient was eligible for root coverage intervention after gingivitis treatment.

### 2.4. Centrifugation Protocol for I-PRF

Protocol for the i-PRF acquisition was performed before surgery, as previously described [15]. Briefly, in the surgical room, a nurse collected the blood from the patient in two plastic tubes that were immediately placed in a centrifuge (Intra-Lock L-PRF Intra Spin, Birmingham, AL, USA) at 2700 rpm and 400 g relative centrifugal force (RCF) for 3 minutes. The yellow portion, the fibrinogen, was aspirated with a syringe.

### 2.5. Surgical Intervention

Gingival recessions were treated by CAF associated with a volume-stable collagen matrix (Fibro-Gide, 20 mm height × 40 mm length × 6 mm thickness, Geistlich Pharma, Wolhusen, Switzerland) functionalized with i-PRF. Briefly, after local anesthesia with 4% articaine containing 1 : 100,000 epinephrine, intra-sulcular incisions with a 15C blade (Swann-Morton, Sheffield, England) were performed, involving the premolars and connected to vertical releasing incisions. A split (interproximal papilla)–full (until mucogingival junction)–split (beyond mucogingival junction) thickness flap was elevated (Figure 1(b)). The soft tissue was mobilized with horizontal incisions in the inner part of the flap, and soft tissue perforations accidentally occurred in the distal region of the right canine and left lateral incisor. Root surfaces were mechanically instrumented with periodontal curettes (Hu-Friedy, Chicago, IL, USA) and chemically treated with 24% ethylenediaminetetraacetic acid (EDTA, Straumann PrefGel, Institut Straumann, Basel, Switzerland) for 2 minutes. Chemical preparation of root surfaces aimed to promote decontamination and improve the blood clot imbrication (adhesion) on the root surface [20, 21]. Interproximal papillae were de-epithelialized. After i-PRF preparation (Intra-Lock L-PRF Intra Spin, Birmingham, AL, USA) [15], a volume-stable collagen matrix was bisected in the long-axis, impregnated with i-PRF for 15 minutes, and positioned in the receptor bed at cementoenamel junction level (Figures 1(c), 1(d), 1(e), and 1(f)). The tension-free flap was coronally positioned 1 mm beyond the cementoenamel junction and sutured (5-0 Vicryl; Ethicon, Johnson & Johnson, Somerville, NJ, USA; Figure 1(g)). The matrix was accommodated on the bed, and no sutures were performed.

### 2.6. Clinical Outcomes and Follow-Up

**Figure 2:**
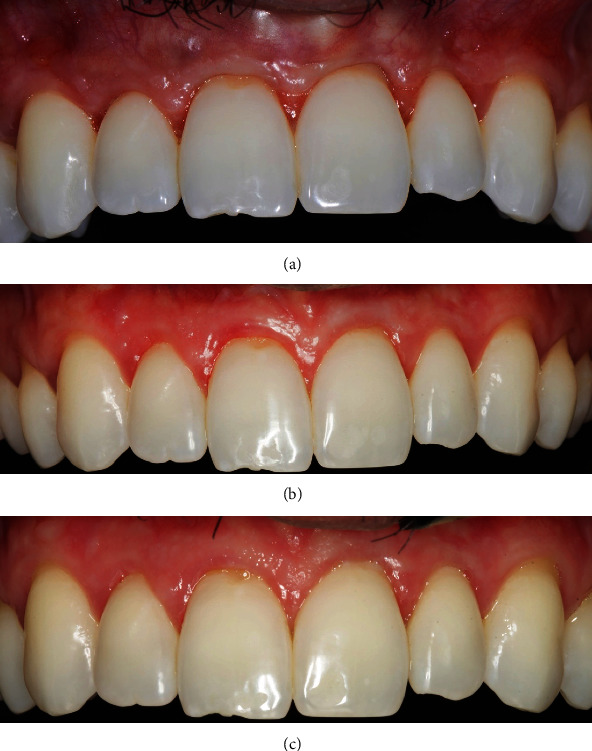
Postoperative follow-up. (a) Clinical view on 2-month follow-up, (b) clinical observation on 9-month follow-up, (c) clinical view on 12-month follow-up.

**Figure 3:**
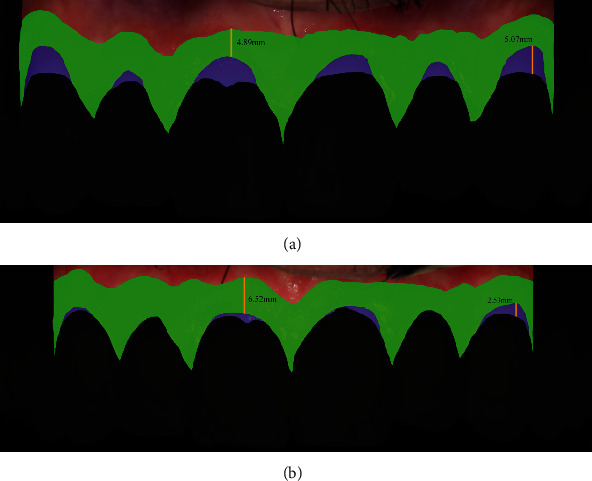
Baseline and 12-month follow-up measurements. (a) Baseline measurements: root recession area (blue); gingival recession height (black line in the blue area); keratinized tissue band (green); keratinized tissue band height (black line in the green area), (b) 12-month follow-up measurements: root recession area (blue); gingival recession height (black line in the blue area); keratinized tissue band (green); keratinized tissue band height (black line in the green area).

A considerable volume increase was observed at the 5-day follow-up, with no signs of inflammation, dehiscence, or necrosis. Sutures were removed 2 weeks after surgery. The surgical area showed a satisfactory healing 2 months after surgery; however, a volumetric soft tissue reduction was detected in comparison with the immediate post-surgical result (Figure 2(a)). More volumetric decrease was observed at the 6-month follow-up. Nevertheless, the gingival margin remained stable, at the same level from the surgical procedure. At the 9-month re-evaluation, probing depth was ≤3 mm in all sites (Figure 2(b)). Gingival margin stability was verified at the 12-month follow-up, with no modification on probing depth, no bleeding on probing, and a stable keratinized mucosa band (Figure 2(c)). Biofilm-induced gingivitis was detected in some teeth at this time. Also, 1 mm of gingival apical migrationat the left canine was observed when comparing the 12-month follow-up with the 2-month follow-up. The patient received additional oral hygiene technique instructions, and gingivitis was treated with non-surgical periodontal therapy involving ultrasonic debridement and professional prophylaxis. Differences from baseline to the 12-month follow-up were calculated for the following measurements: gingival recession area, root coverage area, gingival recession height, root coverage height, and keratinized tissue band height (Figure 3, Table 1).

**Figure 4:**
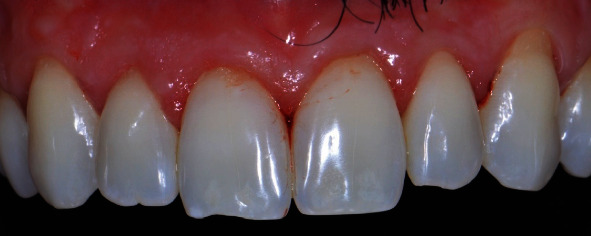
Postoperative follow-up after 30 months. Stable results along the time with minimal modifications. Patient presented bleeding on probing and gingivitis that was further treated during the consultation.

After 30 months of follow-up, clinical parameters were assessed (Figure 4). The probing depths were stable (≤3 mm) in comparison with 12-month follow-up; however, bleeding on probing increased, and the patient was diagnosed with biofilm-induced gingivitis. An additional 1 mm recession was verified at the left canine compared to the 12-month follow-up. Oral hygiene instructions were reinforced, and gingivitis was again treated with ultrasonic debridement and professional prophylaxis.

## 3. Discussion

Gingival recessions treated with autogenous connective tissue graft have been associated with postoperative discomfort in the donor area and restrictions in the graft size, which represents relevant limitations for multiple gingival recession treatment [7]. Xenogeneic substitutes have been employed in regenerative periodontal treatment to overcome those limitations. This case report presented the possibility of using a volume-stable collagen matrix functionalized with i-PRF as an alternative for root coverage of multiple RT1 gingival recessions. The proposed approach demonstrated 83% root coverage of multiple gingival recessions in a 12-month follow-up, with a maintenance of the keratinized mucosa band. If we relate our findings with the literature, other substitutes have a lower percentage of root coverage than the present case report [22].

An additional increase in the gingival recession of the left canine was observed 30 months after root coverage. We suppose this rebound over the treatment outcome occurred due to an inflammatory process associated with a gingivitis condition presented by the patient, who missed the follow-up visits for several months [19]. Despite this, our article is the first to show a 30-month follow-up clinical case on CAF associated with a volume-stable collagen matrix functionalized with i-PRF for multiple root coverage treatment. Those findings suggested that the proposed matrix functionalization is a potential strategy for root coverage in multiple gingival recession cases, with a possible reduction in postoperative morbidity, since no access to a donor area for graft collection was necessary.

The functionalized collagen matrix used in this clinical case intends to mimic the natural extracellular matrix of a connective tissue graft, providing a niche for host cells during healing [23, 24], which allows cell migration, adhesion, and differentiation, as well as supporting vascular proliferation. Moreover, it has been suggested that the volume-stable collagen matrix has long-term dimensional stability due to its low level of cross-linking degradation [25]. An in vitro study showed that i-PRF induced cell migration and increased the gene expression of TGF-*β* mRNA, platelet-derived growth factor (PDGF), and collagen type I [26]. Theoretically, the PRF may also have an epithelial keratinization capacity [27]. Thus, the use of a volume-stable collagen matrix impregnated with i-PRF could increase the healing potential as compared to autogenous connective tissue graft [28–31].

The success of root coverage depends on the gingival thickness and the amount of keratinized mucosa band. A thick soft tissue provides greater tissue stability and reduces the recurrence of gingival recession [32]. Studies reported a complete root coverage in cases with an initial gingival thickness ≤1 mm and a keratinized mucosa band of 2 mm or less when a connective tissue graft was used to augment the soft tissue during root coverage surgery [12, 33]. The present clinical case showed that a satisfactory gingival thickness can be achieved with a functionalized volume-stable collagen matrix with i-PRF, and results were stable for the evaluated period. We speculate that the collagen matrix works as a scaffold for blood clot stabilization, as described in an animal study [34]. The maturation process would result in connective tissue formation with adequate gingival volume. Those findings agree with other studies that prospectively assessed the soft tissue volume increase for long periods [10, 35]. However, our hypothesis requires more studies to be confirmed.

During the follow-up period, a gingival thickness reduction was observed 2 months after the intervention. Matrix thickness was not trimmed before placement in the present clinical case, which may have influenced postoperative results. The large distance between surgical flap and teeth surface may have compromised the vascularization, which could partially explain the incomplete root coverage in some teeth. Another point of consideration was the flap perforations at the right canine and left lateral incisor regions with unpredictable repercussions. Taken together, based on our clinical observations, we suggest that the collagen matrix should be trimmed in its thickness before application on the surgical bed. Other studies also support this recommendation [29, 31].

Considering the limitations of the present case report, it is worth mentioning that, although successful, the proposed approach is limited to one single clinical case. Therefore, our results should be interpreted with caution. Even though a donor site access for connective tissue graft was not necessary in the proposed approach, it is important to highlight that blood collection from the patient in order to produce the PRF is mandatory, which increases morbidity and clinical steps during the surgery. Studies comparing the functionalized collagen matrix with the matrix alone are recommended to draw conclusions on the clinical application of this technique. In addition, this case may have a similar outcome with reduced costs if a CAF alone or in combination with a connective tissue graft was performed [36]. Nevertheless, comparative research would be necessary to evaluate those aspects. In addition, further randomized clinical studies are mandatory to confirm our findings in larger samples. The molecular events and the underlying mechanisms by which collagen matrices associated with blood derivatives influence tissue formation were not elucidated yet.

## 4. Conclusion

Based on the findings of this clinical report and considering that results are limited to one single case, the volume-stable collagen matrix functionalized with i-PRF was a feasible approach for multiple RT1 gingival recession treatment, demonstrating 83% of root coverage in a 12-month follow-up period. Stable results were verified for up to 30 months after root coverage. A donor site access for connective tissue harvesting was avoided, which potentially reduced patient morbidity.

## Figures and Tables

**Table 1:** Measurements of gingival recession area, root coverage area, gingival recession height, root coverage height, and keratinized tissue band height, at baseline and 12 months after the surgical procedure.

	Right canine	Right lateral incisor	Right central incisor	Left central incisor	Left lateral incisor	Left canine
RRAb (mm^2^)	31.13	7.96	38.02	28.87	11.64	32.5
RRAf (mm^2^)	3.18	0	7.18	8.26	0	13.57
RCa (%)	89.94	100	81.11	71.38	100	58.24
GRHb (mm)	5.02	2.09	5.65	3.21	2.6	5.07
GRHf (mm)	0.82	0	1.73	1.33	0	2.53
RCh (mm)	4.2	2.09	3.92	1.88	2.6	2.54
KTBHb (mm)	6.54	6.98	4.89	4.37	5.32	5.85
KTBHf (mm)	6.96	7.32	6.52	5	4.79	4.92
*Δ*KTB (mm)	+0.42	+0.34	+1.63	+0.63	−0.53	−0.93

*RRAb*, root recession area—baseline; *RRAf*, root recession area—final; *RCa*, root coverage area; *GRHb*, gingival recession height—baseline; *GRHf*, gingival recession height—final; *RCh*, root coverage height; *KTBHb*, keratinized tissue band height—baseline; *KTBHf*, keratinized tissue band height—final; *ΔKTB*, keratinized tissue band—difference between initial and final.
